# Clinal variation in natural populations of *Drosophila melanogaster*: An old debate about natural selection and neutral processes

**DOI:** 10.1590/1678-4685-GMB-2023-0348

**Published:** 2024-07-22

**Authors:** Vitória H. Miranda, Rafael Viana Amaral, Rodrigo Cogni

**Affiliations:** 1 Universidade de São Paulo, Instituto de Biociências, Departamento de Ecologia, São Paulo, SP, Brazil.

**Keywords:** Adaptation, biogeographic history, phenotypic plasticity, secondary contact

## Abstract

Distinguishing between environmental adaptations and neutral processes poses a challenge in population genetics and evolutionary studies, particularly when phenomena can be explained by both processes. Clines are genotypic or phenotypic characters correlated with environmental variables, because of that correlation, they are used as examples of spatially varying selection. At the same time, many genotypic clines can be explained by demographic history, like isolation by distance or secondary contact zones. Clines have been extensively studied in *Drosophila melanogaster*, especially in North America and Australia, where they are attributed to both differential selection and various demographic processes. This review explores existing literature supporting this conclusion and suggests new approaches to better understand the influence of these processes on clines. These innovative approaches aim to shed light on the longstanding debate regarding the importance of natural selection versus neutral processes in maintaining variation in natural populations.

## Introduction

Differences in environmental conditions across various locations of a species range can lead to local adaptation. Nevertheless, genetic differences along the species range might stem from alternative processes. Identifying genetic differences that arose from adaptation from those that arose by other, neutral processes is a central question in population genetics and evolutionary studies. Clines are measurable (phenotypic or genotypic) characters that are correlated with environmental gradients, such as altitude and latitude (Endler, 1973). Those gradients are related to abiotic variables, such as temperature and precipitation, and sometimes to biotic variables, such as species richness. They are often employed to tackle the adaptation/neutral processes issue, primarily due to the more predictable gene flow within them (Endler, 1986). However, even in this framework, the difficulties in differentiating neutral from adaptive processes remain.

While specific authors do view sudden shifts in traits (when they align with abrupt changes in the surroundings) as a form of cline (Adrion et al., 2015), the primary usage of the term pertains to describing gradual changes. Phenotypic clines have been extensively studied, especially the ones pertaining to quantitative and physiological tolerance traits, and by virtue of being found commonly across different species, some authors tried to draw rules in order to explain the most frequent patterns. Two major examples of those rules are Bergmann’s rule, which states that individuals of colder environments will be bigger, while individuals of warmer regions will be smaller, and Allen’s rule which states that the surface area/volume ratio of the body for homeothermic animals varies with the average temperature of their habitat (see Jorgensen and Fath, 2008). In the same way, phenotypes related to stress response and reproduction often follow consistent patterns within each category, as populations living in colder, drier habitats, and with higher environmental variation are in general more resistant to stress and generate fewer offspring (Overgaard et al., 2011; Rajpurohit and Nedved, 2013; Adrion *et al.*, 2015; Fabian et al., 2015).

Clines are classically presented as evidence of adaptive evolution (Endler, 1973), yet, correlations between quantitative traits and environmental gradients may result from other evolutionary processes (Keller et al., 2009). For example, phenotypic plasticity may produce seemingly adaptive, phenotypically distinct populations whose genetic differentiation does not align with the distribution of morphological and physiological traits (see Ghalambor et al., 2007). Ultimately, the effect of plasticity can reproduce patterns expected to be the result of selection, as in the case of the freshwater ray-finned fish *Cottus hangiongensis*, where the plastic response to population density produced a clinal variation for life-story traits along the stream of natural rivers (Goto, 1993). Usually, phenotypic characters are measured after rearing the wild-collected specimens in similar favorable conditions and analyzing only the latter generations, so that the genetic variation is assessed. However, it is important to note that this cannot be done in species that cannot be reared on lab conditions or species that have larger generational times. Therefore, evolutionary scientists need to be careful not to believe that clinal traits are necessarily the target of spatially varying selection and account for the role of environmentally-induced phenotypic responses.

Demographic processes may also play a role. Isolation by distance and range expansion of a single funding population may generate a clinal pattern (Excoffier et al., 2009). One example is the clinal variation of the wing shape of North American yellow dung flies, this variation most likely reflects the species’ biogeographic history instead of spatially varying selection, following a pattern of isolation by distance (Schäfer et al., 2018). There are also examples of secondary contact between two invading populations generating a pattern of continuous admixture resembling clines induced by selection. That is the case of the Iberian honeybee, whose genetic clinal variation may have arisen from secondary contact between two divergent lineages (Chávez-Galarza et al., 2015). 

While neutral demographic processes have a role, and because clines, by definition, are linked to environmental gradients, many studies concentrate on the obvious effects of natural selection (e.g. Nielsen et al., 1994; Woods et al., 2012; Kooyers et al., 2015). One fruitful approach to disentangle those processes is the comparison of similar clines across independent, or at least somewhat independent, geographic gradients. Examples would be the comparison between altitudinal and latitudinal clines (Zhang et al., 2019), or different latitudinal clines across the continents (Calfee et al., 2020). Another type of approach could involve using historical samples and analyzing changes in clines over the years. If there were no changes in the environment, selection clines are expected to stay stable. Changes in clinal patterns could point to a more neutral reason for the existence of the clines studied, or that the environment has changed.

Clines have been extensively studied in *Drosophila melanogaster*. This species holds a preeminent position as a model for population genetics studies because of its fast generational time and least challenging survival in lab cultures. It is an African-originated species (David and Capy, 1988; Lachaise et al., 1988) that, through its ancestral dependence on marula fruits, has become a human commensal that spread to all continents (Mansourian et al., 2018). Estimations of the divergence of African and European *D. melanogaster* populations float between 12,800 and 16,800 years ago, depending on the data and type of analyses (Li and Stephan, 2006; Laurent et al., 2011), and those first non-African flies probably originated Asian populations around 2,500 and 5,000 years ago (Laurent *et al.*, 2011). More recently, in the last several hundred years, it invaded North American and Australian continents (David and Capy, 1988; Keller, 2007). Although historical records may suggest a single colonizing event, genomic analysis points to multiple events, at least in North America (Duchen et al., 2013; Kao et al., 2015; Bergland et al., 2016). 

Similar clines in *D. melanogaster* have been described on different continents (Adrion et al., 2015), which by itself may point to a selective cause of this pattern. However, it has been speculated that genomic clines in North America and Australia are, at least in part, the fruit of secondary contact between African and European-related flies (Bergland et al., 2016). This hinders one of the most useful aspects of the study of clines, which is finding targets of natural selection. Genome data remains scarce in places that might help us to better understand *D. melanogaster* targets of differential selection (such as India and South America). Therefore, we review exciting recent research on clinal variation in natural populations of *D. melanogaster* emphasizing different approaches to discern between natural selection and neutral demographic processes as the cause of clinal variation.

## Inferring genetic variation from phenotypic clines

**Figure 1 -:**
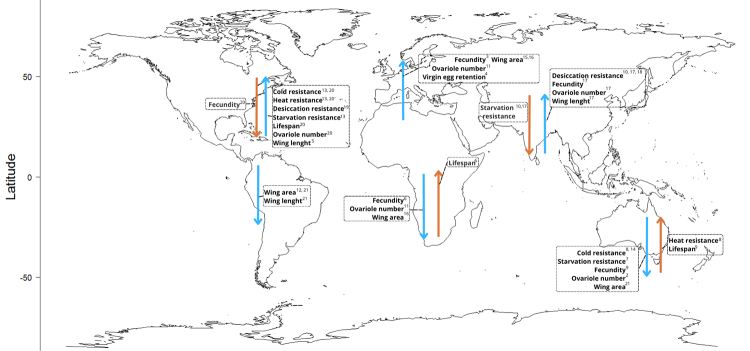
Latitudinal clinal patterns for populations of *Drosophila melanogaster* phenotypes measured at different continental wide gradients. Blue arrows represent a positive correlation with latitude for the phenotype value, while red arrows indicate a negative correlation.

In drosophilids, clinal patterns are represented by a general positive correlation between wing size and fecundity, to latitude, as well as most phenotypes related to stress resistance, like starvation, desiccation, and extreme temperature resistance (Figure 1). If observed independently, such a broad generalization of a pattern could indicate widespread selective pressure. However, because latitudinal variation is multifactorial, and because clinal phenotypes are polygenic, the mechanisms of natural selection underlying phenotypic clines remain to be eluded. Genotype-to-environment interactions, as trade-offs and developmental plasticity, likely have a significant role in the maintenance of the phenotypic variation of populations across a geographical scale (Overgaard et al., 2011).

References: 1) Ayrinhac et al., 2004; 2) Azevedo et al., 1996; 3) Boulétreau-Merle et al., 1982; 4) Boulétreau-Merle *et al.*, 1992; 5) Coyne and Beecham 1987; 6) Fabian et al., 2015; 7) Hoffmann et al., 2001; 8) Hoffmann *et al.*, 2002; 9) Hoffmann *et al.*, 2003; 10) Karan et al., 1998; 11) Klepsatel et al., 2014; 12) Land et al., 1999; 13) Mathur and Schmidt 2017; 14) Overgaard et al., 2011; 15) Pezzoli et al., 1997; 16) Pitchers et al., 2013; 17) Rajpurohit & Nedved 2013; 18) Rajpurohit *et al.*, 2013; 19) Rajpurohit *et al.*, 2018; 20) Schmidt and Paaby, 2008; 21) Zwaan et al., 2000

Thermal plasticity at developmental stages can alter the strength of trait-to-latitude correlations, and thus give insight about adaptation of the populations in their environments. For example, as Rajpurohit et al. (2018) demonstrated a positive latitudinal cline for desiccation tolerance in *D. melanogaster* (Figure 1), the magnitude of differentiation for the trait between the geographic populations increased when the flies were cultured at lower temperatures, where flies developed at 18 ºC were more tolerant than their counterparts developed at 25 ºC and 29 ºC. On another model, investigating wing shape patterns for the yellow dung fly, *Scathophaga stercoraria*, flies from North America, Europe and Japan all presented the same plastic response for rearing temperature; however, a cline was found only for American populations, and the latitudinal north-to-south patterns did not mirror the cold-to-warm plastic responses expected in a scenario of synergy among adaptive plasticity and adaptive genetic divergence (Schäfer et al., 2018). Therefore, to study if plasticity follows the same direction of geographical patterns may help to untangle the challenge of defining adaptive processes behind clines: for the first example in the paragraph, the results are consistent with models of adaptive plasticity in synergy with adaptive genetic divergence, while the second one strengths arguments that processes other than adaptation may explain the found latitudinal cline.

Another key point to consider is that phenotypic clines are not all consistent across all continents, and their inner correlations are prone to change depending on location or experimental procedure. Resistance to desiccation and starvation are in general thought to be correlated (Hoffmann and Harshman, 1999; Rajpurohit et al., 2018), but they are manifested as opposing clines at the Indian subcontinent, as desiccation resistance was not found to have any significant correlation to any climatic variable, while a significant effect of seasonal temperature variation was found to starvation resistance (Karan et al., 1998; Rajpurohit *et al.*, 2013). Also, a lack of reproducibility was found for desiccation tolerance in Australian *D. melanogaster* populations (Hoffmann *et al.*, 2001), and no evidence for cline in starvation resistance was found on the western South American coast (Robinson et al., 2000). Those examples highlight the necessity to study variations within and among populations, as well as different species and different developmental conditions, in order to elucidate the evolutionary shifts at play.

Meanwhile, some correlations are indeed well conserved, like the cline in cold resistance (Gibert et al., 2001; Hoffmann et al., 2002; Ayrinhac et al., 2004; Overgaard et al., 2011). As said previously, the latter was also found to be highly influenced by plasticity, so this may indicate that interactions between different phenotypes and genotypes with the environment are more complex than the individual effects of each might lead to, as reported by Rajpurohit et al. (2013; 2018). Yet, the same authors noted resistance to cold, desiccation tolerance, and body size increase along the geographical latitude. However, they suggested the former two phenotypic categories may be indirect responses to the latter, as body size alone might increase the capacity of flies to take environmental stress; in the same way, higher fecundity rates may demand more individual energy, which reduces the flies’ lifespan, so longevity may not be an adaptive trait as much as a reactive one.

In addition to the possible effects of developmental plasticity and indirect selection, the alignment of seasonal and geographic variations to climate factors signals the role of adaptation in the structure of geographic gradients. Strong and temporally variable natural selection drives rapid and polygenic adaptation of multiple fitness-associated phenotypes on the same time scale as the environmental change and has standing effects after less than four generations (Rudman et al., 2022). The reported speed at which evolution can drive phenotypic change challenges the notion that plasticity should always work as a buffer against environmental change.

Another layer of complexity that can be viewed as phenotypic plasticity, and a promising research avenue, is the influence of interaction with other organisms, especially bacterial symbionts and the microbiome on clinal adaptation. For example, the prevalence of the endosymbiont *Wolbachia* is clinal in natural populations of *D. melanogaster*, and this pattern may not only be involved in protection against virus infection (Pimentel et al., 2020; Cogni et al., 2021), but it may also influence life history and climate adaptation (Strunov et al., 2022). The gut microbiome may also explain part of the life history clinal patterns (Walters et al., 2020). 

## Demographic effects

Because of the range expansion history of *D. melanogaster* and its close relation to humans, many clines in this species might just be the product of its demographic history. This was extensively studied in North America and there were also some studies in Australia. Even so, the resemblance of clines between these continents has often been interpreted as indicative of selection, but it is essential to consider that alternative factors may be at play. The biogeographic history of *D. melanogaster* in North America could help us to better explain many clines.

Although there is historical evidence of a rapid *D. melanogaster* North American range expansion starting from the state of New York and presumably arriving from Europe (Keller, 2007), more ancient reviews (Johnson, 1913; David and Capy, 1988) already advent the possibility of at least another invasion from Africa to tropical America (especially the Caribean islands) through the slave trade. If that were the case, it would mean that North America is a secondary contact zone between African and European populations implying that many of the observed genomic clines in this continent might not be the result of natural selection.

**Figure 2 -:**
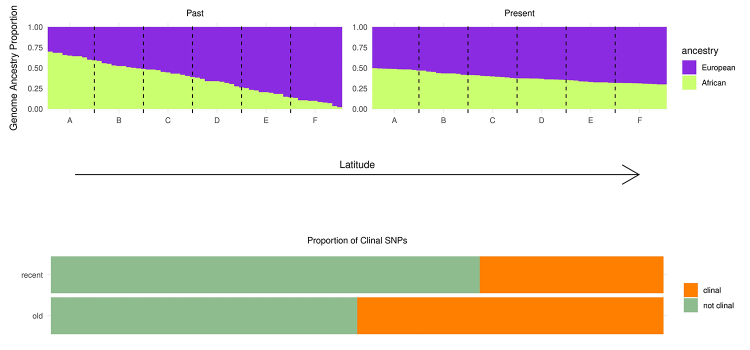
Hypothetical global ancestries of six populations, each with 10 sampled individuals, distributed along the North American latitudinal gradient (A is the furthest southern and F the furthest northern) in the past (left) and present (right). As individuals migrate and admixt global ancestries differences between populations become smaller, and so as the proportion of clinal SNPs (bottom).

Genetic clines of *D. melanogaster* are also very abundant, and it seems unlikely that such an abundance of clines would emerge solely based on spatially varying selection. However, if demographic processes have such an influence, it would be expected for this effect to fade over time as individuals migrate and populations become more admixed (Figure 2). In this scenario, selection would slow this homogenizing process until only the selection-maintained clines remain. Sizable inversions in the *D. melanogaster* genome that span through millions of base pairs may also help to slow homogenization because they suppress recombination on heterozygotes. On some occasions, the whole inversion might be clinal, as is the case of *In(3R)Payne* and *In(2L)t* (Kapun et al., 2016a), and adaptive variations occurring within the inversion may inflate the number of clinal variants due to hitchhiking effects.

Genetic evidence that North American populations are a mix of European and African flies is strong. Analyzing a single population from each continent, North America, Europe, and Africa, Duchen et al. (2013) found that the North American population most likely originated from admixture between African and European populations. They also found an estimated proportion of African ancestry of 15%. When using whole sequencing data from multiple populations in eastern North America it is possible to realize that the African ancestry proportion increases as the latitude decreases, in other words, there is an ancestry cline in eastern North America (Kao et al., 2015; Bergland et al., 2016). This pattern was discernible regardless of the presence of inversions on the analyzed genome. This is an important consideration as most inversions have an African origin, which could be the sole reason for this pattern. Moreover, there are higher levels of linkage disequilibrium in North American populations than in the African or European continents, which might indicate a recent introgression (Kao *et al.*, 2015). 

The pattern of ancestry proportions in Australia is similar to the one in North America. Australian populations closer to the equator have more African ancestry than Southern populations (Bergland et al., 2016). However, the genomic data quality for those populations was worse than the ones for North America, and the natural history of *D. melanogaster* in that continent was not so well documented. Nevertheless, when analyzing multiple populations around the world it was inferred that the Australian population was most likely formed by migration from both Africa and Europe, and the African proportion in that population was inferred to be around 33% (Arguello et al., 2019). 

In spite of the similarities in patterns, Australian *D. melanogaster* biogeographical history is likely to be different from what happened in the American continent. Throughout the 19th century, most ships that arrived on the Australian coast were British. It is possible those ships had been ported in Africa and India before their arrival in Australia, and thus already brought mixed flies. This would mean that Australia is not a secondary contact zone, as in North America, and that rapid ecological sorting might have created the ancestry clines (Bergland et al., 2016). Indeed, it would be a huge coincidence if both colonizations had followed the exact same pattern, which does not seem likely. It would be interesting, however, to see if this ancestry cline is present in less studied continents, like South America. If it does, that would cast doubt over the biogeographic history explanation for the ancestry clines, and another explanation for this pattern would be needed, like ecological sorting or selection.

## Evidence of selection

Traditionally, phenotypes naturally related to temperature, desiccation resistance, and photoperiod are also usually linked to selection. This is reasonable, as those factors vary greatly along latitudinal gradients and are thought to be the primary agents of spatially varying selection. Molecular and genetic clines were also interpreted as signs of natural selection, mainly because their variation is not influenced by the environment and because they are not as complex as phenotypic characters. Classical examples involve the study of allozymes, such as the ones present in 6-phosphogluconate dehydrogenase (*Pgd*) and glucose-6-phosphate dehydrogenase (*G6pd*). The convergence of latitudinal clines in those loci was promptly interpreted as the product of selection (Oakeshott et al., 1983; Berry and Kreitman, 1993). 

One effective approach involved comparing the slope of clines derived from known neutral markers such as microsatellites with those of quantitative phenotypic traits (Gockel et al., 2001). The approach relied on the premise that if the slope of phenotypic traits diverged from that of neutral markers, it would suggest the influence of spatial differential selection. Such a discrepancy was noted in Australia between body size and microsatellite data (Gockel *et al.*, 2001). With genomic studies, it was possible to scan the whole genome and look for evidence of selection linked to environmental gradients. One of the first papers to use whole genome sequencing was Fabian et al. (2012), in which they used pool-seq in three populations across North America’s East Coast. They observed an overrepresentation of coding SNPs among the top 0.5% most differentiated SNPs between the extremes of the cline and identified a list of candidate genes that may be involved in clinal adaptation. 

The clinality of *D. melanogaster*’s genomic inversions can also be the product of natural selection. This might happen because there may be adaptive variation inside the inversion and, as there is lower recombination in the inversion zone, the hole inversion might get hitched. Another possible explanation is that those inversions might keep together a set of alleles that work epistatically contributing to local adaptation. In North America, at least two major inversions are thought to be clinal due to spatially varying selection, *In(3R)Payne* and *In(2L)t* (Kapun et al., 2016a). *In(3R)Payne* is present in both North American and Australian latitudinal clines and was related to body size, a classical phenotypical cline (Kapun *et al.*, 2016b; Rako et al., 2006). Moreover, its clinal intercept increase over 20 years was linked to climate change in Australia (Anderson et al., 2005; Umina et al., 2005), but (Kapun *et al.*, 2016a) found it to have the same slope and intercept between 1970 and 2010 in North America. On the other hand, *In(2l)t* is a seasonally clinal inversion, that is, it has a clinal slope in the fall, but in summer it becomes almost homogeneous along the latitudinal gradient (Kapun *et al.*, 2016a). 

Another strategy to identify clinal variants under selection is the recognition of patterns across similar changes in the environment. For example, *D. melanogaster* phenotypes vary across seasons, especially in temperate regions (Behrman et al., 2015). Given that temperature is likely the primary selection factor across latitudes, the correlation between clinal and seasonal changes can provide evidence of clinal adaptation. Molecular and genomic variation across seasons were described (Bergland et al., 2014), and replicated field experiments were able to link this variation to selection (Rudman et al., 2022). A more concrete example is the *cpo* gene, which is known to affect the diapause trait in *D. melanogaster*. Clinal variants in this gene are also strongly seasonal, and the selection coefficient associated with those seasonal changes was estimated to be quite large (s = 0.241-0.59) (Cogni et al., 2014). Furthermore, Cogni *et al.* (2015), examined 128 SNPs within 46 metabolic genes and observed a pattern where alleles that were more frequent at higher latitudes also exhibited high frequencies immediately after winter, at the start of spring, and that their frequencies decreased as seasons went by. Using whole genome sequencing and comparing samples collected along the North American latitudinal gradient and six pairs of seasonal samples, Rodrigues et al. (2021) found that the correlation between seasonal and clinal variants was stronger within coding regions. This is interesting because it is coherent to expect that functional regions would present a more pronounced effect of selection. 

In an important study, Machado et al. (2016) compared *D. melanogaster* clines with clines from its sister species, *D. simulans*. Overall, *D. melanogaster* clines were much more abundant, which in principle, could be explained by the difference between the biogeographical history of the species. However, clines in *D. melanogaster* were also more stable over the analyzed years than clines in *D. simulans*. This could indicate that a selection-migration balance was reached in *D. melanogaster*. Moreover, functional genic classes were enriched for clinal SNPs, which also indicates that clinal variants are under selection. They also looked for shared clinal genes between the species and found that those shared genes were enriched for temperature-dependent expression genes in both species.

Another approach to disentangle selection from demographic processes is using historical samples and comparing them to recent ones. That happens because if clines are really maintained by selection and if there are no major environmental changes, clines are expected to stay stable. The diapause trait, for example, is clinal across the North American latitudinal gradients (Schmidt et al., 2005) and it is strongly linked to the *cpo* gene which carries many clinal SNPs. When samples from 1997 and 2009-2010 were compared, those SNP clines were stable, that is, there were no shifts nor changes in the slopes of those clines (Cogni et al., 2014). On the other hand, the instability of clines can simply mean that the environment changed during the analyzed period. This holds particularly valid when considering the impact of climate change. For instance, the alcohol dehydrogenase (*Adh*) locus has one of the most studied latitudinal clines, the frequency of its allozyme variant AdhS increases towards Ecuador in North America and Australia (Oakeshott et al., 1982). When Umina et al. (2005) compared *AdhS* allele frequencies across the Australian latitudinal gradient from 1979-1982 to 2002-2004, they found that the cline had shifted toward the northern-related (warmer-related) allele and there were no changes in the slope of the cline. This shift was credited to climate change. However, analyzing the AdhS allele, Cogni *et al.* (2017) found that this cline was stable in North America from 1883-1988 to 2009-2010. In the same paper, Cogni *et al.* (2017) compared clines from 1997 to 2009-2010 of 21 SNPs in 15 metabolic genes, many of them remained unchanged across the years, but some clines were lost or gained while others had their slopes changed. Comparing whole genome sequences from historical and recent samples could provide important insights into the patterns of change. 

Lange et al. (2022) conducted a study where they analyzed 65 lineages collected between 1975 and 1983 from Rhode Island and compared them to recent samples. They found a decrease in the global African-related ancestry, and when they looked at clinal SNPs, they found that Northern-related alleles had their frequencies increased. Those results are unexpected as global ancestry should become more similar along the latitudinal gradient as migration brings more African-related variants to the North and European-related variants to the South. Moreover, Rhode Island experienced a 1 ºC increase in temperature over that period and it would be reasonable to assume that the Southern-related allele (warmth-related allele) would be the one to increase in frequency. Those results could be explained by asymmetric migration but also by directional selection driven by something else than temperature. In this study, they did a Gene Ontology (GO) enrichment analysis on population branch statistics (PBS) outliers (using the old samples and recent fall and spring samples). They found that resistance to insecticide and nervous system were enriched GO categories. Although very insightful, this paper only looks at a single population along the cline, the same pattern may not repeat itself in other positions along the North American latitudinal gradient, and it would be interesting to compare recent and historical samples across the gradient.

Basically, two types of change in clines can occur: there can be a shift change when the frequency of a given allele arises along the whole cline; or there can be a slope change, when the frequency changes in only part of the gradient, weakening or strengthening the cline (Rodrigues and Cogni, 2021). Shifts are not expected if the primordial reason for the existence of the clines had been demographic history. Still, shifts are expected to be a product of climate change, especially if the shift happens toward the warm-related allele (the southern-related allele in the Northern hemisphere and the northern-related allele in the Southern hemisphere). On the other hand, changes in slope are harder to understand. Demographic history can explain the weakening of the slopes but so could climate change-driven selection. Using whole genome sequence tools on historical samples can help us see the general patterns of clinal change, we could look for regions enriched to a certain type of clinal change, for example. We could also look for changes in global and local ancestry. If clines were mainly caused by demographic history, migration would weaken the clines and populations would present more similar rates of the same ancestry, moreover, this would happen along the whole genome. 

## Conclusion

In this review, we analyzed different potential explanations for the existence of *D. melanogaster* latitudinal clines. There are two main explanations for the existence of clines. The first is spatially varying selection, as the environments become progressively more different along the latitudinal gradient so do the selective forces. The second one takes into account the biogeographical history of *D. melanogaster* in relatively newly colonized continents. The use of temporal samples could help us to disentangle demographic and selective processes. There is compelling evidence for both explanations and most probably they both contribute to the clinal phenomenon. The real question should be which loci are more influenced by selection. 

When examining clinality using phenotypical characteristics, the complexity deepens. The multifactorial nature of this kind of data makes all evolutionary studies harder. Despite being generally associated with fitness, the phenotypes exemplified in this review do not vary universally across the whole distribution of species. This not only challenges the old ideas of general rules associated with phenotypic clines but also shows the importance of seeking different locations to better test whatever hypothesis we may have to explain the pattern found in a particular cline. For drosophilids, this means a need to rely on more than just the study of North American and Australian *D. melanogaster* natural populations, the majority in regards to the prevalence of latitudinal clines, and enhance the mapping of variation for populations of all continents to elucidate if demography, plasticity, and adaptation all generate the observed geographic variation. Genomics studies and the understanding of adaptive tracking (Rudman et al., 2022) are also important to reveal how the phenotypes are reacting to selective pressure over them, as the described traits are also in general polygenic, and thus likely influenced by pleiotropy and intergenic interactions (Rajpurohit and Nedved, 2013).

In conclusion, in the years to come, we expect to see exciting new studies on sequencing genomes of historical samples, clinal patterns in different continents, advances in the difficult task of mapping phenotypic and molecular variation, and new developments on the influence of symbionts and the microbiome on clinal adaptation. The comparison of different time points and the study of clinal patterns in unstudied continents (e.g. South America) are promising approaches that will bring light to the long debate about the importance of natural selection and neutral processes in maintaining variation in natural populations. 
